# Fanconi Syndrome Associated with Valporic Acid: A Case Report

**Published:** 2011-11-01

**Authors:** R Shiari, L Bagherzade, M R Alaei

**Affiliations:** 1 Mofid Children’s Hospital, Shahid Beheshti University of Medical Sciences, Tehran, Iran; 2 Pediatrics Infectious Research Center (PIRC), Shahid Beheshti University of Medical Sciences, Tehran, Iran

**Keywords:** Fanconi syndrome, Valporic acid, Myopathy, Carnitine

Dear Editor,

Fanconi syndrome is the most common cause of proximal renal tubular acidosis (RTA) in children and is characterized by hypophosphatemia due to phosphaturia, renal glucosuria (glucosuria with a normal plasma glucose concentration), aminoaciduria, and normal anion gap metabolic acidosis. Muscle weakness is one of the Fanconi syndrome manifestations.[1][2]

Valporic acid (VPA) is an anti-epileptic drug widely used to treat partial and generalized seizures especially in children. Fanconi syndrome is a rare side effect in children during long-term VPA treatment.[3][4] Herein, we reported a 14-year-old Iranian boy with progressive muscle weakness. He was diagnosed as a case of Fanconi syndrome secondary to VPA administration and showed dramatic response to treatment by carnitine supplement.

The 14-year-old Iranian boy was referred to Mofid Children’s Hospital because of progressive muscle weakness. He was treated with VPA for 2 years due to his seizures attacks. With the exception of epilepsy, he was a healthy and active boy with normal development and no relevant findings in his past medical history. Almost one year after he was started on VPA, he developed progressive muscle weakness with myalgia and gradually he became unable to walk. On physical examination, muscle atrophy was detected and his proximal and distal muscle strength in both upper and lower limbs decreased. His Laboratory findings were as follows:

Metabolic acidosis (VBG=pH 7.14, PCo2 = 41.9 mmHg, serum bicarbonate=12.3 mmol/L), hypophosphatemia (p=3.2 mg/dL), hypokalemia (k=2.47 mEq/dL) with normal calcium (9.2 mg/dL). The patient also had proteinuria (1+), glucosuria (1+) and his urine pH was 6, which were compatible with Fanconi syndrome. Blood urine nitrogen and creatinine levels were 3 mg/dL and 0.9 mg/dL respectively. His parathormone hormone level was normal. Random sample of urine showed urine phosphorous of 131.4 mg/dL, urine calcium=186 mg/dL, and urine cratinine=29 mg/dL.

Kidney ultrasound revealed hyperechogenicity in both kidneys and his muscle biopsy was highly compatible with mitochondriopathies or carnitine deficiency. Because VPA appears to be the first suspicion for renal involvement, it stopped and carnitine was administered. Within 3 months, the muscle weakness improved and his kidney function recovered. People treated with VPA experienced some side effects; however, it is widely used as an effective antiepileptic drug. One of the important side effects of VPA is acquired type of Fanconi syndrome.

A PubMed-based review of the literature revealed that Fanconi syndrome was a rare side effect in children during VPA treatment. However, the detailed clinical characteristics of this disease remained unclear.[5] On the other hand, prolonged treatment with VPA enhanced renal losses of carnitine esters, lowers serum carnitine level, and resulted in secondary carnitine deficiency.[6] Secondary carnitine deficiency was described with some disorders including Fanconi syndrome and VPA therapy.[6][7]

Herein, we presented a 14-year-old Iranian boy that his physical examination and laboratory data were compatible with Fanconi syndrome. Considering 2 years history of VPA consumption, there was a probability of “VPA induced Fanconi syndrome”. Therefore, VPA was discontinued. Since carnitine deficiency has been seen after long term VPA therapy, L carnitine started for our patient.[8] His response was dramatic and all of his symptoms resolved within 6 months.

Up to now there are 13 cases reported as “VPA induced Fanconi syndrome'”. A comprehensive review of these cases, in 2010 by Endo et al.,[9] brought beneficial points. The average age of patients was 8.2 years, the duration of VPA administration was 6.4±3.4 months and almost all of the 13 patients were severely disabled, bed ridden, and fed through a gastrostomy tube. VPA was discontinued and urinary abnormalities disappeared within 3.8±2.7 months. All patients had favorable renal outcome.[10]

In contrast to this review article, our patients were mentally and developmentally healthy and VPA was started due to recent attacks of seizure. Similar to other cases, marked clinical improvement was observed after cessation of VPA therapy but there was a unique point knowing that carnitine deficiency as a following sequence of VPA therapy, L Carnitin was started. The astounding response confirmed our finding. So we can conclude that clinician should be aware of Fanconi syndrome as a result of prolonged VPA administration, although it is uncommon.
